# Sequence variation in the microRNA region of a cancer virus

**DOI:** 10.18632/oncotarget.26471

**Published:** 2018-12-18

**Authors:** Joseph M. Ziegelbauer

**Affiliations:** Joseph M. Ziegelbauer: HIV and AIDS Malignancy Branch, Center for Cancer Research, National Cancer Institute, National Institutes of Health, Bethesda, MD, USA

**Keywords:** KSHV, HHV8, microRNA, SNP

There are many diseases associated with latent viral infections. However, targeting latent viral infections remains a challenge for treatment. One of the difficulties is that there are few viral gene products that are expressed during latency to target with inhibitors. Therefore, it is important to understand the sequence variations in latent viral gene products. These sequence variations could affect the expression patterns of latent viral gene products and prevent certain sequence variants from being targeted by an antiviral molecule. In this issue, Marshall et al. dive deeper into answering these questions by analyzing genetic variation (single nucleotide polymorphism, SNPs) and expression of latently-expressed viral microRNAs (miRNAs).

Previously, this group had investigated the correlations of SNPs, expression, and disease in the context of Kaposi’s Sarcoma Herpesvirus (KSHV) infection and multicentric Castleman’s disease (MCD). In this latest report, they extend these studies in the context of another KSHV-associated disease, Kaposi’s sarcoma skin lesions from South African and American biopsies. Many of these newly discovered SNPs in the miRNA region were specific to the various KSHV subtypes. In a similar finding to their MCD study, they found the most variation in KSHV microRNA regions, miR-K12-8 and miR-K12-9. Umbach et al. [1] and the research team led by Denise Whitby had previously shown [2] that miR-K12-9 is not expressed in a patient-derived KSHV-infected cell line and this was due to sequence changes that disrupt the biogenesis of that miRNA. Thus, these results highlighted the connection between sequence variation and mature miRNA expression.

Following the detection of many new SNPs in the miRNA region of KSHV, this new report also investigated the potential impacts of these sequence variations on predicted structures and mature miRNA expression levels in the same clinical samples. Specific structural formations are necessary for normal RNA binding protein associations and miRNA biogenesis steps. The authors discovered that K12-8-3p and miR-K12-9-5p were expressed at lower levels in samples from South Africa, compared to American samples. In contrast, overall the expression levels of the majority of KSHV miRNAs were higher in the South African samples, despite having similar KSHV loads in the American samples. Additionally, they found SNPs in the mature miRNA regions of miR-K12-5, miR-K12-6, miR-K12-9, and miR-K12-10. Alterations of a single base with a mature miRNA can dramatically alter the mRNAs that are targeted by that specific miRNA. An interesting SNP (A121617G) near the start of pre-miRNA K12-3 was found in all of the South African samples and almost half of the American samples. This SNP correlated with expression changes in seven different mature miRNAs, suggesting more distant structural changes and changes in RNA-binding protein interactions that altered the mature miRNA expression pattern. These authors noted that some SNPs were discovered at sites near the 5’ end of the pre-miRNAs, which are important sites for association with the miRNA processing factors DGCR8 and Drosha. Structural predictions suggested that many of these SNPs would not disrupt the majority of miRNA hairpin structures.

These new results present many interesting ideas. For example, how many of these SNPs directly correlate to changes in miRNA expression in experimental cell culture systems? The SNPs detected in MCD samples were previously tested in this fashion [2]. With more samples, information about which SNPs strongly correlate with KSHV-associated diseases could lead to new diagnostic tools. Much of this research has been focused on sequence variation and biogenesis of these viral miRNAs, but understanding if SNPs alter the association with miRNA degradation proteins like MCPIP1/Regnase [3] will deepen the understanding of miRNA regulation. A recent report described sequence variations in KSHV genomes from 45 individuals [4]. This genome-wide analysis did not find that the microRNA region was the most variable region in these 45 samples, but it will be interesting to determine if SNPs outside of the region analyzed by Marshall et al. may also influence expression of latent proteins and non-coding RNAs. Integration of sequence variations, expression of viral genes (proteins and non-coding RNAs), alterations of human gene expression, and clinical outcomes may lead to new biomarkers, diagnostic assays, and specific therapies to combat viral infection-associated diseases.

**FUNDING**

This research was supported by the Intramural Research Program of the NIH, NCI. The funder had no role in study design, data collection and analysis, decision to publish, or preparation of the manuscript.
